# Tobacco and alcohol use among adolescents and young adults in aspirational districts in India: NFHS-5 based secondary analysis

**DOI:** 10.11604/pamj.2025.51.17.46828

**Published:** 2025-05-15

**Authors:** Shiv Kumar Mudgal, Vipin Patidar, Suresh Kumar Sharma, Rakhi Gaur, Ramesh Kumar Huda, Jayvardhan Singh, Saurabh Varshney

**Affiliations:** 1 College of Nursing, All India Institute of Medical Sciences, Deoghar, Jharkhand, India; 2 College of Nursing, All India Institute of Medical Sciences, Jodhpur, Rajasthan, India; 3 Indian Council of Medical Research-National Institute for Implementation Research on Non-Communicable Diseases, Pali Rd, Bhagat Ki Kothi, Jodhpur, Rajasthan 342005, India; 4 All India Institute of Medical Sciences, Deoghar, Jharkhand, India

**Keywords:** Alcohol, adolescents, aspirational districts, prevalence, tobacco, young adults

## Abstract

**Introduction:**

an early initiation of alcohol and tobacco use is associated with addiction and significant health complications. This study aimed to determine the prevalence and associated factors of tobacco and alcohol usage among adolescents (15-19 years) and young adults (20-24 years) in India's aspirational districts.

**Methods:**

data from India's biggest demographic and health survey, the National Family Health Survey-5 (NHFS-5), 2019-21, were reviewed. Alcohol and tobacco (in any form) usage were measured using the self-reported binary responses. Multivariate binary logistic regression was used to examine relationships with different demographic variables.

**Results:**

among the 47,343 individuals, tobacco usage was substantially higher in men than women (adolescents: 15.9% versus 1.2%; young adults: 31.8% versus 2.2%). The prevalence of alcohol usage was also higher among men (adolescents: 8.1% versus 0.4%; young adults: 21.8% versus 1.0%), with statistical significance (p < 0.0001) for all sex-related differences. Use of tobacco and alcohol was significantly associated with illiteracy, male gender, and young adults. A higher prevalence was observed among Christians in rural areas and lower socio-economic settings.

**Conclusion:**

the results highlight the necessity of targeted efforts by appropriate stakeholders and governmental agencies to lower alcohol and tobacco usage, particularly among vulnerable populations. More research is required to determine the root causes of these habits and the related health risk behaviours.

## Introduction

The steadily rising proportion of tobacco and alcohol use has demonstrated that alcohol and tobacco usage are no longer exclusive to rich countries or urban settings [1,2]. Tobacco smoking is expected to kill more than eight million consumers, while alcohol consumption is another substantial risk factor for early mortality and disability among those aged 15-49 years. These statistics account for up to 10% of all deaths in this age category [3,4], and a majority of those who use tobacco reside in India and other countries in South East Asia [5,6]. Moreover, the South-East Asia Region is estimated to have the highest average rate of tobacco use compared to all other WHO regions, at around 29% in 2020, which is higher than the global prevalence (22.3%) [2].

In 2022, an estimated 253 million individuals aged 15 and older used tobacco products in India. Regarding tobacco use, the country ranks second worldwide and first in the WHO South-East Asia Region [7]. The most recent National Family Health Survey (NFHS-5), which was carried out between 2019 and 2021, indicates that most Indians face a significant burden of tobacco and alcohol, which could possibly be more prevalent in the aspirational districts [8]. Previous research done in India found that low economic level, limited educational attainment, and living in rural areas were all contributing to tobacco and alcohol consumption in adolescents and young adults [9-11]. It is important to note that these studies focus mostly on the overall demographic trends in the country. The association between tobacco and alcohol use and poor socio-economic status, which is common among teenagers and young people in aspirational districts, emphasizes the importance of investigating tobacco and alcohol use in these areas [12,13].

The government of India established the “aspirational districts” program in 2018. These districts have weak development indices, high poverty rates, and restricted access to healthcare facilities, which increases their likelihood of using tobacco and alcohol. These districts were chosen because of their poor performance on a variety of development indices, including health, education, nutrition, and basic infrastructure [14]. There are manifold reasons for targeting the 15-24-year age group. Firstly, research has shown that adolescents are especially vulnerable to engaging in health-risk choices such as tobacco and alcohol use [15]. Tobacco use often starts during adolescence, with a majority of persons who currently smoke tobacco having initiated before age 21 [16]. It is essential to comprehend the elements that contribute to tobacco and alcohol use in this population to develop preventative strategies that can lower the probability of chronic addiction and related health risks. Secondly, this age group may be especially vulnerable to social and cultural pressures that influence tobacco usage. Cultural standards, peer pressure, and societal expectations all impact how people behave in their teens and early adult years. An analysis of these components can highlight particular challenges these age groups face. Thirdly, there are specific risks associated with alcohol and tobacco use for people of this age. Studies conducted on this population revealed that drinking alcohol during adolescence is linked to numerous negative consequences, including, for example, psychological distress [17], poorer academic performance [18], risky sexual behaviour [15], suicidal behaviour [19], smoking, appetite changes, weight loss [20], headaches, liver damage, and sleep disturbance [16]. Also, adolescent alcohol use can have long-term health consequences that may last until adulthood, such as adult alcohol dependency and addiction, diminished work capacity [21], diabetes, mental health complications [22], and premature death [23]. Finally, the public health policy can target 15-24-year-olds as a single group because of common biological, developmental, and socio-economic contexts of use and similar challenges faced. Previous research in the aspirational districts primarily focused on economic status, child and maternal health, and academic achievement, with no studies aimed at tobacco and alcohol use in these age groups, given that it is a vital health concern prevalent among them. As a result, this study aimed to examine the prevalence and determinants of tobacco and alcohol use using data from the National Family Health Survey-5 (NHFS-5), 2019-21.

## Methods

**Source of data:** we utilized data from a nationally representative household-based survey (NFHS-5) carried out in India between 2019 and 2021. A total of 707 districts across 28 Indian states and 8 union territories were part of the study, with 636,699 houses and 2,843,917 individuals successfully interviewed. The NFHS-5 used a stratified two-stage sampling technique. In the initial phase, the sampling procedure was conducted differently in urban and rural regions within each district. Villages were chosen using probability proportional to size (PPS) as primary sampling units (PSUs) in rural regions, while census enumeration blocks (CEBs) were chosen using PPS systematic sampling as PSUs in urban areas. Following the thorough mapping and household listing of the chosen PSUs, 22 houses were randomly chosen using systematic random sampling in each of the rural and urban clusters selected for the second stage.

**Study sample:** the Indian government introduced the “aspirational districts” initiative in 2018. The label of “aspirational districts” was applied to 112 districts from 27 Indian states. These districts included 19 from Jharkhand, 13 from Bihar, 10 from Odisha and Chhattisgarh, 8 from Madhya Pradesh and Uttar Pradesh, and 7 from Assam. Andhra Pradesh and Telangana each have three districts, while Rajasthan and Maharashtra have five and four districts, respectively. There are also two districts in Gujarat, Karnataka, Punjab, Tamil Nadu, Uttarakhand, and Jammu and Kashmir. Meanwhile, Arunachal Pradesh, Haryana, Himachal Pradesh, Manipur, Meghalaya, Mizoram, Nagaland, Sikkim, and Tripura all contribute one district to the program. In NFHS-5, 825954 adults and adolescents were interviewed, and 778611 of these participants were dropped because they lived in non-aspirational districts of India and were older than 24 years. The study's data analysis included 47343 adolescents and young adults (ages 15-24).

**Study Variables:** dependent variables include the current use of tobacco and alcohol in any form. Tobacco in any form, including cigarettes, smoking pipe tobacco, cigars, water pipes, hookah, sniffing tobacco products, eating gutkha, pan masala with tobacco, khaini, and other types of chewable tobacco products. Alcohol used included Tadi Madi, country liquor, beer, and wine. Current use was defined by use during the last month. Smoking, tobacco and alcohol usage were binary variables with an outcome of 0 and 1 recorded as 'No' and 'Yes'. Independent variables were selected after a detailed literature review. The demographic variables included were age (15-19 versus 20-24), religion (Hindu, Muslim, Christian, and Others), location of residence (urban versus rural), education status (illiterate, primary, secondary, higher), and wealth index (poorest, poor, rich, richer, and richest) were grouped into relevant categories.

**Data analysis:** we used SPSS (Statistical Package for the Social Sciences) version 25.0. The estimates were a weighted percentage with a confidence interval at 95%. The association across both dependent and independent variables was assessed with the chi-square test. The study applied a multivariable logistic regression model to explore the social determinants of alcohol and tobacco use in the study population. Independent variables with p values <.02 were considered for the multivariable logistic regression model, and the backwards likelihood ratio method was utilized to determine the best-fit model. We calculated the adjusted odds ratio (aOR) to calculate the effects of social determinants of alcohol and tobacco use. Statistical significance was defined as a p-value of <.05.

**Ethics statement:** NFHS-5, 2019-21 obtained ethical approval from the International Institute for Population Sciences (IIPS), located in Mumbai. The ICF International Review Board (IRB) additionally reviewed and approved the survey for ethical purposes. Interviews were conducted only after every participant gave their consent. The NFHS-5, a publicly accessible anonymous dataset that cannot be used to identify survey participants, is hosted on the webpage of the Demographic and Health Surveys (DHS) Program, so institutional ethics approval was not necessary. Although we received an official letter of authorization from DHS allowing us to use the specified survey datasets for this research.

## Results

Tobacco and alcohol use were found to be more prevalent in adolescent males residing in Indian aspirational districts. In a total of 47343 participants (5089 males and 42254 females), 20.3% of males and 1.3% of females were using any form of tobacco. Similarly, 12% of males and 0.7% of females also consumed alcohol.

**Socio-demographic information:** the socio-demographic background of male participants showed that the majority, 54.8%, were in their late adolescence (15 to 19 years). Regarding the kind of education, 72.2% of them had finished the secondary education level, 13.5% finished higher education and only 7.2% of the total participants are illiterate. A greater number (83.6%) of males lived in rural areas with Hinduism (77.2%) being the most prominent religion, followed by Islam (13.3%), Christianity (5.1%) and others (4.4%). Out of all the participants, 34.9% fell under the category of poorest, 26.4% under poor, 18.2% took the middle place and only 13% and 7.5% were reported as richer and richest groups, respectively. The demographic characteristics of female participants revealed that late adolescents made up 51.9%. As for secondary education, completed females stood at 67.8%, while females who finished higher education reached 11.3%. The illiterate women made up 12% of the analysed population. A greater number of females (85.9%) also lived in rural areas, with Hinduism (75.7%) being the notable religion, followed by Islam (15.4%), Christianity (5%), and other religions (3.9%). Based on the wealth index indicator, 37.8% fell within the poorest group, while 26.3% belonged to the poorer group. From this population, middle categories were 17.7%, richer women were 11.9%, and lastly, 6.4% came from the richest classification.

**Figure 1:**
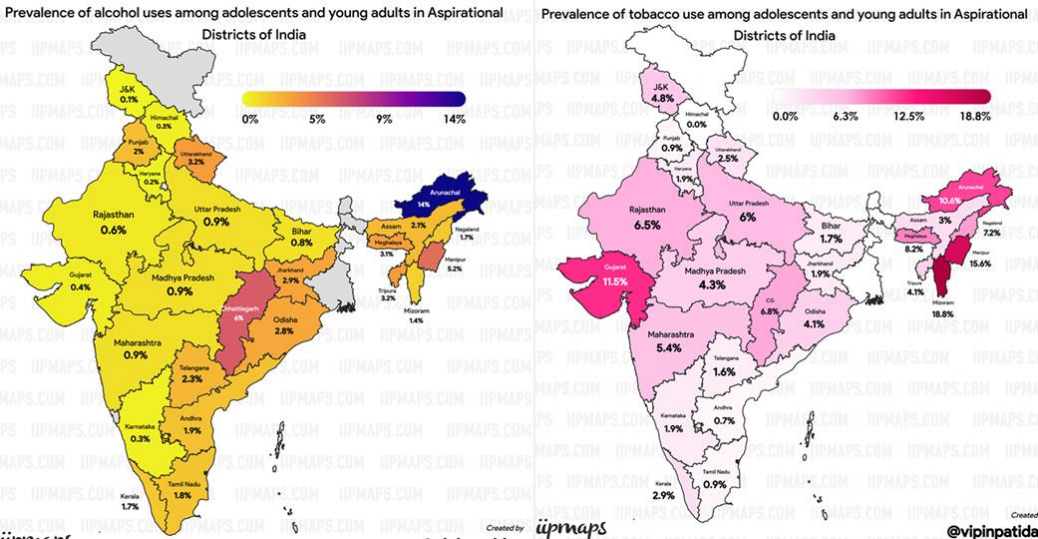
prevalence of tobacco and alcohol use among adolescents and young adults in aspirational districts of India, NFHS-5 (2019-2021)

**Regional prevalence of tobacco and alcohol use:** the regional segregation (Figure 1) shows that the range of prevalence of tobacco and alcohol use across Indian states was 0% to 18.8% and 0.1% to 14%, respectively. Interestingly, the usage was found to be highest in the population residing in the northeastern region. The highest prevalence of tobacco use was found in Mizoram (18.8%), Manipur (15.6%), and Gujarat (11.5%). Similarly, the highest prevalence of alcohol use was found in Arunachal Pradesh (14%), Chhattisgarh (6%) and Manipur (5.2%).

**Association of demographic traits with tobacco and alcohol usage:** the prevalence and association of different demographic traits and tobacco and alcohol usage in adolescents and young adults are displayed in Table 1. In both groups, the prevalence of tobacco usage was significantly higher in men than in women. (late adolescents: 15.9% versus 1.2%; young adults: 31.8% versus 2.2%). Similarly, the prevalence of alcohol usage was substantially greater in men (late adolescents: 8.1% versus 0.4%; young adults: 21.8% versus 1.0%), and all sex-based differences were statistically significant (p < 0.0001). Higher levels of educational background were associated with a decrease in alcohol and tobacco use (p < 0.0001). Among the illiterate group of people, 7.1% of late adolescents and 6.7% of young adults use tobacco, whereas just 1.0% and 2.4% of those with a higher educational background do similarly.

**Table 1:** prevalence of tobacco and alcohol use across demographic variables in aspirational districts of India, NFHS-5 (2019-2021)

Demographic variables	Tobacco use				Alcohol use			
	Late Adolescent n (%)	Late Adolescent χ^2^ (p)	Young adult n (%)	Young adult χ^2^ (p)	Late Adolescent n (%)	Late Adolescent χ^2^ (p)	Young adult n (%)	Young adult χ^2^ (p)
**Sex**								
Male	444 (15•9)	1896•61 (0•000)	731 (31•8)	3702•92 (0•000)	225 (8•1)	1197•66 (0•000)	501 (21•8)	2970•35 (0•000)
Female	271 (1•2)		439 (2•2)		81 (0•4)		202 (1•0)	
**Education**								
Illiterate	139 (7•1)	197•23 (0•000)	232 (6•7)	112•49 (0•000)	54 (2•8)	74•80 (0•000)	155 (4•5)	56•20 (0•000)
Primary	101 (5•3)		167 (7•5)		48 (2•5)		91 (4•1)	
Secondary	465 (2•3)		663 (5•3)		193 (1•0)		378 (3•0)	
Higher	10 (1•0)		108 (2•4)		11 (1•1)		79 (1•8)	
**Type of residence**								
Urban	68 (2•0)	11•62 (0•001)	140 (4•1)	8•685 (0•003)	28 (0•8)	5•736 (0•017)	86 (2•5)	4•235 (0•040)
Rural	647 (3•0)		1030 (5•3)		278 (1•3)		617 (3•2)	
**Religion**								
Hindu	547 (2•9)	31•81 (0•000)	880 (5•1)	40•95 (0•000)	235 (1•3)	98•44 (0•000)	594 (3•4)	107•67 (0•000)
Muslim	81 (2•1)		158 (4•7)		4 (0•4)		14 (0•4)	
Christian	64 (5)		101 (9•0)		34 (2•7)		45 (4•0)	
Others	23 (2•3)		31 (3•4)		33 (3•3)		50 (5•5)	
**Wealth index**								
Poorest	402 (4•1)	107•72 (0•000)	529 (6•7)	97•83 (0•000)	187 (1•9)	65•53 (0•000)	377 (4•8)	117•54 (0•000)
Poorer	180 (2•8)		324 (5•5)		61 (0•9)		150 (2•5)	
Middle	85 (2•0)		191 (4•6)		40 (0•9)		96 (2•3)	
Richer	36 (1•3)		86 (2•9)		12 (0•4)		45 (1•5)	
Richest	12 (0•9)		40 (2•4)		6 (0•4)		35 (2•1)	

n; number of participants, χ^2^; chi-square.

Alcohol use followed a similar pattern, with the highest prevalence among the illiterate and the lowest among the highly educated in both males and females. Prevalence rates of alcohol and tobacco use were higher in rural regions compared to urban areas. Tobacco use in rural and urban areas was 5.3% versus 4.1% among late adolescents and 3% versus 2% among young adults respectively. Similarly, alcohol consumption followed this pattern, showing statistically significant differences related to residential status (p < 0.05). Significant differences (p < 0.0001) were observed in alcohol and tobacco use between religious affiliations. Christians had the highest rates of tobacco use (late adolescents: 5.0%; young adults: 9.0%) and alcohol use (late adolescents: 2.7%; young adults: 4.0%), while Muslims had the lowest rates, especially for alcohol use (late adolescents: 0.4%; young adults: 0.4%). The group with the lowest socio-economic status had the highest prevalence rates of alcohol use (late adolescents: 1.9%; young adults: 4.8%) and tobacco use (late adolescents: 4.1%; young adults: 6.7%), while the group with the highest socio-economic status had the lowest incidence rates (p < 0.0001).

**Determinants of tobacco and alcohol use:** the determinants of tobacco and alcohol use among adolescents and young adults in aspirational districts of India (Table 2). The odds of tobacco use in any available form were significantly higher in the young adults (aOR=2•286, CI=2•054-2•544) compared to the adolescents. Similarly, females had lower odds of tobacco use (aOR=0•43, CI=0•39-0•48) than males. The participants had a lower odds ratio of tobacco use as their education level increased, with higher education having the lowest odds (aOR=0•189, CI=0•148-0•240). Religious differences were pronounced, with Christians having the highest odds ratio of tobacco use (aOR=1•953, CI=1•619-2•357) compared to individuals from other religions. The wealthier groups showed a consistent decrease in tobacco use, with the richest having the lowest odds (aOR=0•368, CI=0•268-0•505).

**Table 2:** logistic regression between tobacco and alcohol use with demographic variables in aspirational districts of India, NFHS (2019-2021)

Demographic variables	N;47343, Male;5089, Female; 42254	
	Tobacco use	Alcohol use
	aOR (95%CI)	aOR (95%CI)
**Age**		
15-19®	1	1
20-24	2•286 (2•054-2•544)	3•259 (2•804-3•788)
**Gender**		
Male®	1	1
Female	0•43 (0•39-0•48)	0•29 (0•25-0•33)
**Education**		
Illiterate®	1	1
Primary	0•855 (0•713-1•027)	0•747 (0•582-0•959)
Secondary	0•406 (0•352-0•469)	0•340 (0•280-0•412)
Higher	0•189 (0•148-0•240)	0•236 (0•176-0•317)
**Type of residence**		
Urban®	1	1
Rural	0•969 (0•816-1•151)	0•872 (0•691-1•100)
**Religion**		
Hindu®	1	1
Muslim	0•829 (0•711-0•966)	0•091 (0•056-0•147)
Christian	1•953 (1•619-2•357)	1•512 (1•167-1•960)
Others	0•684 (0•508-0•920)	2•236 (1•719-2•909)
**Wealth index**		
Poorest®	1	1
Poorer	0•800 (0•706-0•907)	0•548 (0•458-0•655)
Middle	0•688 (0•589-0•803)	0•550 (0•445-0•682)
Richer	0•446 (0•359-0•553)	0•327 (0•240-0•444)
Richest	0•368 (0•268-0•505)	0•370 (0•254-0•538)

®Reference, aOR; adjusted odd ratio, CI; Confidence interval, N; total number of participants.

The odds of alcohol consumption were significantly higher in young adults (aOR=3•259, CI=2•804-3•788) compared to adolescents. Females again had significantly lower odds of alcohol use (aOR=0•29, CI=0•25-0•33) than males. Similar to tobacco use, education played a substantial role in reducing alcohol use, with those having higher education (aOR=0•236, CI=0•176-0•317) having lower odds of alcohol consumption. The people from other religions show higher odds of alcohol use (aOR=2•236, CI=1•719-2•909). The wealthier individuals had progressively lower odds of alcohol use, with the richest having the lowest (aOR=0•327, CI=0•240-0•444).

**Figure 2:**
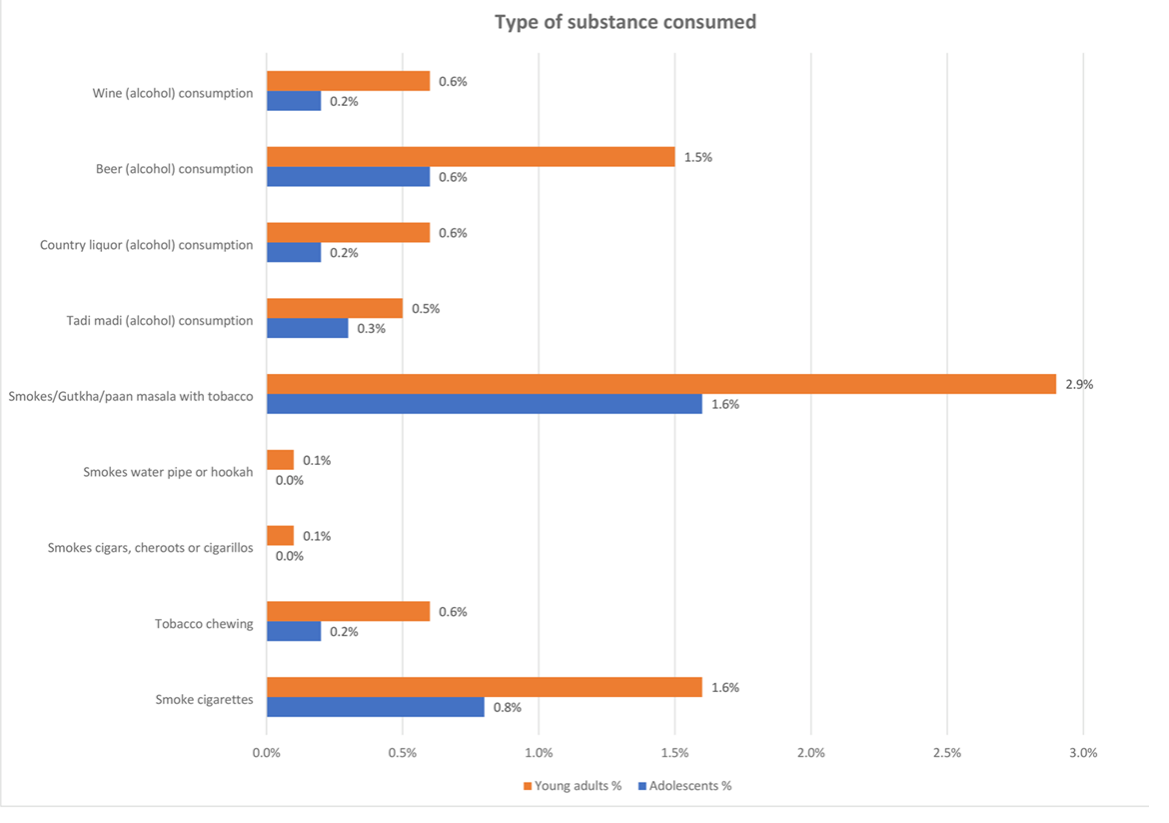
type of tobacco and alcohol consumed by adolescent and young adult participants in aspirational districts of India, NFHS (2019-2021)

**Uses of various forms of tobacco and alcohol:** Figure 2 illustrates the various forms of tobacco and alcohol consumed by adolescents and young adults residing in India's aspirational districts. Data indicated that young adults reportedly consumed more in all categories than adolescents surveyed. Results revealed that among adolescents, the most widely used tobacco and alcohol forms were tobacco-containing Gutkha/paan masala (1.6%), cigarette smoking (0.6%), and beer (0.6%). Among young adults, the most widely used tobacco and alcohol forms were tobacco-containing Gutkha/paan masala (2.9%), cigarette smoking (1.6%), and beer (1.5%). Other forms of tobacco and alcohol use, such as chewing tobacco, smoking cigars, smoking hookah, drinking wine, and drinking country liquor, remained more common but were less widespread among young adults than among adolescents.

## Discussion

To the best of our knowledge, this study was the first to assess the prevalence and examine the factors influencing tobacco and alcohol use in adolescents (15-19 years of age) and young adults (20-24 years of age) using NHFS-5 data from a nationally representative household sample in India's aspirational districts. This analysis highlights the factors that drive any type of tobacco (smoked and/or smokeless) and alcohol use, with a special emphasis on the social, educational, and demographic aspects that affect tobacco and alcohol use.

**Prevalence of tobacco and alcohol use:** the study revealed that the prevalence of tobacco use among adolescent males and females residing in aspirational districts was 15.9% and 1.2%, respectively. These rates were higher than the national prevalence among Indian adolescents of the same age group, as reported by NFHS-5 data, which documented tobacco use at 14.3% among males and 0.8% among females. A comparable pattern was also observed among young adults. Similarly, the prevalence of alcohol usage in adolescent males and females in aspirational districts was 8.1% and 0.4%, respectively, exceeding the national prevalence rates of 5.8% and 0.2% for males and females of the same age group, as reported in NFHS-5 data. This trend was likewise reflected in young adult populations.

**Association and determinants of tobacco and alcohol use:** the survey reveals a significant gender gap in use of alcohol and tobacco, with prevalence rates for all forms of tobacco and alcohol use being much higher in men than women. This gender disparity is in line with earlier research showing that, especially in socio-economically deprived areas, men are typically more prone to participate in risky conduct, such as tobacco and alcohol use [24,25]. The low prevalence of tobacco and alcohol usage in women may be supported by social and cultural norms that dissuade women from engaging in such behaviours.

Educational attainment emerges as a pivotal determinant in reducing the likelihood of tobacco and alcohol use. Adolescents and young adults with higher educational levels show significantly lower odds of smoking, tobacco use, and alcohol consumption. This aligns with global findings that education acts as a protective factor against tobacco and alcohol abuse by enhancing awareness about the associated health risks and providing better economic opportunities, reducing the stressors that could result in tobacco and alcohol use [26-28].

socio-economic status as reflected in the wealth index, also plays an important role in tobacco and alcohol use patterns. The analysis reveals that individuals from poorer economic backgrounds are more likely to engage in smoking, tobacco, and alcohol use. This trend is consistent with the theory that economic hardship can lead to increased stress and a higher chance for tobacco and alcohol use as a coping mechanism [27,29]. The findings suggest a need for targeted interventions in economically disadvantaged communities to address the root causes of tobacco and alcohol usage.

Religious affiliation significantly influences alcohol and tobacco usage; Christian males are more likely than people of other religions to smoke, use tobacco, and drink alcohol. Cultural variations in social norms and the acceptance of tobacco and alcohol use in religious communities could be the cause of this discrepancy [27]. It underscores the importance of culturally sensitive interventions that take into consideration the specific beliefs and practices of different religious groups. In addition, the data demonstrate that living in a rural region is associated with higher rates of tobacco and alcohol use than living in an urban region. This might be due to the less strict enforcement of tobacco and alcohol control laws in remote areas and restricted access to healthcare and educational resources [29].

The direct association between tobacco use and socio-economic disadvantages reflected by lower education, income, and scheduled tribe status has been revealed in the previous studies from India and the global north [30,31]. Higher tobacco use in the disadvantaged group might be due to targeted marketing strategies, positive social norms, life stress, or social networks of tobacco users [2,26]. More worryingly, individuals from underprivileged backgrounds are less likely to be given support for tobacco cessation; thus, they are more likely to suffer from severe use disorders and experience higher health burdens due to tobacco use, including higher risks of lung cancer and cardiac disorders [9,27]. Therefore, public health measures targeting adolescents and young people to prevent the initiation of tobacco use become essential.

**Strengths and limitations:** one of the primary strengths of this research is its use of the nationally representative dataset from the NFHS-5, which provides a robust sample size and allows for generalizability of the findings to the broader population of Aspirational Districts in India. The comprehensive methodology employed by NFHS-5, including stratified two-stage sampling, enhances the consistency and validity of the data. Additionally, the study's focus on Aspirational Districts, which are commonly characterized by poor development indicators and higher levels of poverty, provides valuable insights into the socio-economic and cultural determinants of tobacco and alcohol use in these underprivileged areas.

Despite its strengths, the study also has several limitations. First, the cross-sectional manner of the NFHS-5 data limits the ability to draw causal inferences between the identified determinants and tobacco and alcohol use. Second, the reliance on self-reported data for tobacco and alcohol use may introduce reporting bias, particularly underreporting due to the social stigma associated with tobacco and alcohol use, especially among females. Third, the study doesn't take into consideration possible confounding variables like mental health status, peer influence, and exposure to media and advertisements promoting tobacco and alcohol use, which might be important in influencing tobacco and alcohol use behaviours. Lastly, while the study focuses on aspirational districts, it does not delve into the specific interventions or policies that have been put into practice in these districts to curb tobacco and alcohol use.

## Conclusion

The prevalence of alcohol and tobacco use among adolescents and young adults in India's Aspirational Districts is higher than the national averages, which highlights a serious public health issue. The study offers comprehensive evidence that educational background, socio-economic level, gender, religion, and rural residency significantly influence tobacco and alcohol usage in adolescents and young adults in India's Aspirational Districts. The focused public health initiatives that address these factors are vital for reducing tobacco and alcohol usage and achieving the health-related Sustainable Development Goals (SDGs). Future studies ought to concentrate on the effectiveness of such interventions and explore the underlying psychosocial factors contributing to tobacco and alcohol use in these regions.

### What is known about this topic

- Tobacco and alcohol use remain a substantial public health challenge in the 21^st^ century;
- India is ranked second globally and first in South-East Asia in tobacco consumption.

### What this study adds

- Tobacco usage among those aged 15-24 years was 20.3% for males and 1.4% for females;
- Alcohol use was 12% for males and 0.7% for females.

## References

[1] World Health Organization (WHO) Tobacco.

[2] Verma M, Rana K, Bhatt G, Sharma N, Lal P (2023). Trends and determinants of tobacco use initiation in India: analysis of two rounds of the Global Adult Tobacco Survey. BMJ Open.

[3] Njie GJ, Kirksey J C, Jacques N, Adetokun A, Ross J, Owens A (2023). Changes in Tobacco Product Use Among Students Aged 13 to 15 Years in 34 Countries, Global Youth Tobacco Survey, 2012-2020. Prev Chronic Dis.

[4] Smith L, López Sánchez GF, Pizzol D, Oh H, Barnett Y, Schuch F (2024). Global Trends in the Prevalence of Alcohol Consumption Among School-Going Adolescents Aged 12-15 Years. J Adolesc Health.

[5] Benzi IMA, Stival C, Gallus S, Odone A, Barone L (2023). Exploring Patterns of Alcohol Consumption in Adolescence: the Role of Health Complaints and Psychosocial Determinants in an Italian Sample. International Journal of Mental Health and Addiction.

[6] World Health Organization (WHO) Alcohol.

[7] World Health Organization (WHO) The tobacco industry is targeting the youth.

[8] International Institute for Population Sciences (IIPS) ICF (2022). National Family Health Survey (NFHS-5), 2019-21: India.

[9] Thakur JS, Prinja S, Bhatnagar N, Rana SK, Sinha DN, Singh PK (2015). Widespread inequalities in smoking & smokeless tobacco consumption across wealth quintiles in States of India: Need for targeted interventions. Indian J Med Res.

[10] Siahpush M, Singh GK, Jones PR, Timsina LR (2010). Racial/ethnic and socioeconomic variations in duration of smoking: results from 2003 2006 and 2007 Tobacco Use Supplement of the Current Population Survey. J Public Health (Oxf).

[11] Donaldson SI, Dormanesh A, Perez C, Majmundar A, Allem JP (2022). Association Between Exposure to Tobacco Content on Social Media and Tobacco Use: A Systematic Review and Meta-analysis. JAMA Pediatr.

[12] Ladusingh L, Dhillon P, Narzary PK (2017). Why Do the Youths in Northeast India Use Tobacco?. J Environ Public Health.

[13] Martin LT, Haas A, Schonlau M, Derose KP, Rosenfeld L, Rudd R (2012). Which literacy skills are associated with smoking?. J Epidemiol Community Health.

[14] NITI Aayog Transformation of aspirational districts: a new India by 2022.

[15] Irwin CE (2010). Young adults are worse off than adolescents. J Adolesc Health.

[16] Thompson AB, Tebes JK, McKee SA (2015). Gender differences in age of smoking initiation and its association with health. Addict Res Theory.

[17] Delker E, Brown Q, Hasin DS (2016). Alcohol Consumption in Demographic Subpopulations: An Epidemiologic Overview. Alcohol Res.

[18] Nadkarni A, Tu A, Garg A, Gupta D, Gupta S, Bhatia U (2022). Alcohol use among adolescents in India: a systematic review. Glob Ment Health (Camb).

[19] Weinstein ND, Marcus SE, Moser RP (2005). Smokers' unrealistic optimism about their risk. Tob Control.

[20] Salvi D, Nagarkar A (2018). A qualitative study exploring women's journeys to becoming smokers in the social context of urban India. Women Health.

[21] Goel S, Kar SS, Verma M, Sivanantham P, Naik BN, Gupta D (2021). Evidence on article 5.3 of FCTC (tobacco industry interference in tobacco control activities) in India-a qualitative scoping study. BMC Public Health.

[22] Feeny E, Dain K, Varghese C, Atiim GA, Rekve D, Gouda HN (2021). Protecting women and girls from tobacco and alcohol promotion. BMJ.

[23] Jakob J, Joss S, Meier AN, Tal K, Schoeni A, Marti J (2022). The price of nicotine dependence: A comparison of the cost of nicotine across products in Switzerland, Germany, USA, Sweden, France and the UK, in 2019. Tob Prev Cessat.

[24] Singh A, Ladusingh L (2014). Prevalence and determinants of tobacco use in India: evidence from recent Global Adult Tobacco Survey data. PLoS One.

[25] Singh PK, Yadav A, Singh L, Singh S, Mehrotra R (2020). Social determinants of dual tobacco use in India: An analysis based on the two rounds of global adult tobacco survey. Prev Med Rep.

[26] Pattojoshi A, Tikka SK (2020). School-based substance use disorder prevention in India: A brief appraisal. Indian J Psychiatry.

[27] Jose J, Chaudhary A, Ghosh A, Goel S (2025). Youthful Choices: A Secondary Analysis of the NFHS-5 Data to Examine Tobacco Use in Indian Adolescent Girls and Young Women. Indian J Psychol Med.

[28] Mudgal SK, Nath S, Chaturvedi J, Sharma SK, Joshi J (2022). Neuroplasticity in Depression: A Narrative Review with Evidence-Based Insights. Psychiatr Danub.

[29] Jadnanansing R, Blankers M, Dwarkasing R, Etwaroo K, Lumsden V, Dekker J (2021). Prevalence of substance use disorders in an urban and a rural area in Suriname. Trop Med Health.

[30] Garrett BE, Martell BN, Caraballo RS, King BA (2019). Socioeconomic Differences in Cigarette Smoking Among Sociodemographic Groups. Prev Chronic Dis.

[31] East KA, Reid JL, Rynard VL, Hammond D (2021). Trends and Patterns of Tobacco and Nicotine Product Use Among Youth in Canada, England, and the United States From 2017 to 2019. J Adolesc Health.

